# Lower Reproductive Rate and Lamb Survival Contribute to Lower Lamb Marking Rate in Maiden Ewes Compared to Multiparous Ewes

**DOI:** 10.3390/ani12040513

**Published:** 2022-02-18

**Authors:** Dayna Hutchison, Bronwyn E. Clarke, Serina Hancock, Andrew N. Thompson, Elise Bowen, Caroline Jacobson

**Affiliations:** Centre for Animal Production and Health, Murdoch University, South Street, Murdoch, WA 6150, Australia; 32901095@student.murdoch.edu.au (D.H.); bronwyn.clarke@murdoch.edu.au (B.E.C.); s.hancock@murdoch.edu.au (S.H.); andrew.thompson@murdoch.edu.au (A.N.T.); elisejbowen@gmail.com (E.B.)

**Keywords:** lamb survival, scanning rate, sheep reproduction, maiden ewe

## Abstract

**Simple Summary:**

The reproductive performance of ewes in their first breeding season (maiden ewes) can be poorer and more variable than at subsequent breeding seasons. However, the extent and causes of the poorer reproductive performance of maiden ewes on Australian sheep farms are not well understood. We used a survey of Australian sheep farmers to compare the reproductive performance of maiden ewes in their first breeding season to multiparous ewes (ewes that have been bred two or more times) on the same farms. We found that the difference in lamb marking rate between non-Merino ewe lambs and multiparous ewes on the same farm was 58%, and for maiden Merino two-tooth ewes, the difference in marking rate compared to multiparous ewes was 22% lower. Poorer reproduction in maiden ewes was due to a combination of poorer reproductive success to mid-pregnancy (reproductive rate), plus poorer survival of lambs between mid-pregnancy and lamb marking. Reproductive performance for maiden Merino two-tooth ewes was correlated with multiparous ewes on the same farm, whereas the reproductive performance of non-Merino ewe lambs was more variable and not associated with the reproductive performance of their multiparous counterparts. The reproductive efficiency of maiden ewes could be improved by addressing factors that improve the reproductive rate and lamb survival between scanning and lamb marking.

**Abstract:**

Suboptimal reproductive performance of maiden (primiparous) ewes remains a source of inefficiency for the Australian sheep industry. However, the extent and causes of the poorer reproductive performance of maiden ewes on Australian sheep farms are not well understood. Here, we show the reproductive performance of maiden ewes relative to their multiparous counterparts on the same farms across Australia using a cohort survey. The difference in marking rate for non-Merino maiden ewe lambs compared to multiparous ewes was 58% (74 vs. 132%; *p* < 0.001), and this was attributable to a 50% difference in reproductive rate (109 vs. 159%; *p* < 0.001) and 16% difference in lamb survival to marking (67 vs. 83%; *p* < 0.001). The difference in marking rate for maiden Merino two-tooth ewes lambing at approximately 2 years-of-age compared to mature multiparous ewes was 22% (80 vs. 102%; *p* < 0.001) and this was attributable to a 24% difference in reproductive rate (108 vs. 132%; *p* < 0.001) and 3% difference for lamb survival (75 vs. 78%; *p* < 0.05). Positive correlations for reproduction traits (reproductive rate, lamb survival and marking rate) between maidens and multiparous ewes were observed for maiden Merino two-tooth ewes (*p* < 0.001), but these correlations were weak or non-existent for non-Merino ewe lambs. Strategies to improve both reproductive rate and lamb survival can address the poorer and more variable reproductive performance of maiden ewes.

## 1. Introduction

Improving the reproductive performance of maiden (primiparous) ewes lambing for the first time at either approximately 12 months of age (ewe lambs) or 24 months of age (two-tooths) has been identified as a priority for the Australian sheep industry [1]. It is widely accepted that the reproductive performance of maiden ewes is generally poorer and more variable compared to multiparous ewes. However, the reproductive performance of maiden ewes on commercial farms in Australia has not been well quantified. Marking rate, which describes the number of lambs marked (tailed and/or tagged) relative to the number of ewes joined (mated) to rams, has increased in Australia by about 15% over the last 30 years [2,3]. This has been attributed to changes in flock structure and greater adoption of husbandry practices, including differential management of single- and multiple-bearing ewes to optimise the condition score [4,5]. However, industry data for marking rates are based predominantly on mature multiparous and mixed-age ewes and do not differentiate between maidens and multiparous ewes [2,3]. Improved understanding of the reproductive performance of maiden ewes can inform benchmarks for reproduction and strategies aimed at improving whole-flock reproductive performance. Apart from the economic benefits from improved reproductive performance, improving ewe and lamb survival can improve animal welfare and address societal demands for ethical livestock production [6].

Marking rate is a function of reproductive rate or the number of foetuses identified at pregnancy scanning during mid-pregnancy and foetal and lamb survival between scanning and lamb marking. A range of factors is known to contribute to lower marking rates in ewe lambs compared to multiparous ewes, including lower fertility and ovulation rates, higher embryo loss between conception and scanning and lower foetus and lamb survival between scanning and marking (reviewed by Kenyon et al. [7]). There has been less work on the performance of ewe lambs in Australia compared to New Zealand. Thompson et al. [8] and Clune et al. [9] recently reported considerable variation in reproductive rate and or marking rates between different flocks of ewe lambs. In these studies, the average reproductive rate was 108%, and foetal and lamb losses between scanning and marking exceeded 35%. Lamb mortality in the perinatal period was the primary cause of lamb mortality after scanning, although abortions were an important contributor in some flocks [9]. However, neither study compared the performance of ewe lambs and multiparous ewes or focussed on the relative contributions of reproductive rate and foetus and lamb survival to the poorer and more variable marking rates for ewe lambs.

Surprisingly, even less is known about the reproductive rate and lamb survival for maiden two-tooth ewes compared to multiparous ewes. A large study on 43 farms over 4 years in South Australia found that reproductive rate was approximately 13% lower for maiden two-tooth ewes, and survival of both single and twin lambs was approximately 10% lower compared to multiparous ewes on the same farm [10]. Other work has reported no significant differences in lamb survival between maiden two-tooth ewes and multiparous ewes across eight flocks around Australia over five years [11]. Lockwood et al. [12] reported no differences in lamb survival between maiden two-tooth ewes and multiparous ewes from survey respondents in south-eastern Australia, whereas, in New Zealand, survival was reported to be 6% lower for maiden two-tooth ewes than mature multiparous ewes. Whilst differences in lamb survival between maiden two-tooth ewes and mature multiparous ewes may be less than observed between maiden ewe lambs and multiparous ewes, even small differences impact national marking rates due to the large number of maiden two-tooth ewes joined annually.

This study aimed to determine the difference in reproductive performance between maiden and multiparous ewes across major sheep producing regions of Australia to inform strategies to improve reproductive performance in maiden ewes. We hypothesised that (i) maiden ewes joined either as ewe lambs or two-tooth ewes will have lower marking rates than multiparous ewes, and (ii) this will be due to a combination of lower reproductive rate and lower lamb survival between scanning and marking.

## 2. Materials and Methods

### 2.1. Study Design and Setting

This study surveyed sheep producers from Western Australia, South Australia, Victoria, New South Wales and Tasmania. Sheep producers were recruited for the survey between 2019 and 2021 and completed a questionnaire focused on reproductive performance for ewes that lambed between 2018 and 2020. This included data recorded at pregnancy scanning (typically conducted 70–90 days from the start of mating period) and lamb marking (tail docking).

### 2.2. Study Participants

Sheep producers were contacted by the project team via phone or e-mail. Contact details for producers were obtained through their participation in other sheep reproduction studies, or from commercial providers of sheep pregnancy scanning and livestock production advisors. Livestock production advisors and pregnancy scanning providers also contacted clients on behalf of the project team to confirm interest and eligibility for participation before providing contact details to the project team. The survey was also promoted to sheep producers using social media and in newsletters by sheep breeders’ societies, sheep production extension organisations, grower groups and livestock production advisors.

Participants were provided with a cover letter outlining the aims of the project, requirements for eligibility to participate and information relevant for providing informed consent to participate. Respondents were selected for inclusion in the survey on the basis that: (a) they separately managed maiden ewes mated as ewe lambs (7–10 months at start of mating period) or two-tooth ewes (16–22 months at start of mating period), (b) utilised pregnancy scanning by transabdominal ultrasonography to determine the number of foetuses for maiden and multiparous ewes and (c) were able to determine lamb survival to marking for maiden and multiparous ewes on the same property, which generally required managing maiden ewes separately from multiparous ewes during lambing. Very few responses were received for Merino ewe lambs and non-Merino maiden two-tooth ewes during the first 12 months of the study, so they were subsequently excluded from the survey, and only non-Merino ewe lambs and Merino two-tooth ewes were targeted thereafter.

The most common reasons for exclusion from study were: (a) the number of foetuses was not determined for some or all of the maiden or multiparous ewes, and/or (b) maiden ewes were mixed with multiparous ewes during lambing and thus lamb survival to marking could not be determined separately for maiden and multiparous ewes.

### 2.3. Questionnaire and Measurements

The questionnaire could be completed by the producer either as a hard copy or electronically, or was completed by the research team via a telephone interview with the producer. Follow-up contact with producers was made via telephone, text message or e-mail, where only partial data had been returned. Pre-testing of the questionnaire was done initially with research staff at Murdoch University and then with farmers participating in other sheep production projects to ensure that the questionnaire layout and questions were clear and unambiguous. Modifications to the question design were made in response to feedback during the pre-testing phase. Further refinements to questions were made throughout the study in response to feedback from respondents and following preliminary analysis of the data that informed the statistical methods.

The questionnaire included four questions about general farm details, including location, farm size and the number of Merino and non-Merino ewes on the farm. The remainder of the questionnaire was divided into three sections that included reproductive data for (a) maiden ewes at scanning, (b) maiden ewes at lamb marking and (c) multiparous ewes at scanning and lamb marking. Sheep data were collected for each mob of maiden ewes and for the total population of multiparous ewes for each farm. Data collected for maiden ewes at pregnancy scanning included the number of ewes mated, ewe age, ewe breed, breed of ram used, month of mating period, body condition score at mating, number of ewes scanned, number of foetuses identified at scanning and number of non-pregnant (dry) ewes. Maiden ewe data collected at lamb marking included mob or paddock name, number of ewes, pregnancy status (single, twin, triplet or mixed), month lambing commenced, condition score at lambing, number of lambs marked and number of ewe deaths (if known). Data for multiparous ewes included the number of ewes, ewe breed, breed of rams used, number of ewes scanned, number of foetuses identified at scanning, number of non-pregnant ewes, number of lambs marked and number of ewe deaths (if known).

Respondents had the option of providing additional data outlining the management of maiden ewes, including the length of the mating period, average condition score at mating and lambing, predominant pasture types and feed-on-offer and supplementary feeding strategies. Condition score was reported on a scale of 1 (very thin) to 5 (very fat), as previously described [13]. For feed-on-offer and condition score, there was a question asking if respondents had measured or estimated these values and whether they had previously received training in performing these measurements.

Validity of the sample size for respondents was assessed using an assumption of 10% expected difference in mean for reproductive parameters (traits), expected standard deviation of 20 and power of test 80. Based on this, 90% confidence intervals could be determined with 36 responses and 95% confidence intervals determined with 49 responses.

### 2.4. Quantitative Variables

Reproductive rate (%) for maiden and multiparous ewes were determined based on the number of foetuses identified at pregnancy scanning expressed relative to the number of ewes scanned. Lamb survival (%) was determined based on the number of live lambs present at lamb marking expressed relative to the number of foetuses identified at scanning. Marking rate (%) was determined based on the number of live lambs present at marking expressed relative to the number of ewes joined. Ewe mortality (%) was determined based on the number of ewe deaths between pregnancy scanning and lamb marking expressed relative to the number of ewes that were pregnant at scanning (i.e., excluded ewes not pregnant at scanning).

### 2.5. Statistical Analysis

Farm location was coded into categories based on the Australian Bureau of Agricultural and Resource Economics (ABARE) region that are based on agricultural profile [2]. Statistical analyses were performed using GENSTAT (VSN International 2017, Hemel Hempstead, UK) and IBM SPSS Statistics (version 24) (IBM 2021, Armonk, NY, USA). Reproductive traits (reproductive rate, lamb survival and marking rate) for maiden and multiparous ewes were compared initially using a paired sample *t*-test (two-tailed), including a 95% confidence interval of the difference. The assumption of normality of the difference between maiden and multiparous ewes for reproductive traits were tested using a Shapiro–Wilk test. This indicated that the assumption of normality for the difference was not met for reproductive rate and ewe mortality (*p* < 0.001). Subsequently, comparisons of reproductive traits between maiden ewes and multiparous counterparts on the same farm were compared using a two-tailed non-parametric related-samples Wilcoxon signed-rank test. Correlations between reproductive traits for maiden and multiparous ewes on the same farm were determined using bivariate Pearson correlation (two-tailed) and linear regression.

Association between farm, sheep and management factors with reproductive performance traits were analysed with linear mixed-effects models at farm level and mob/paddock level. Models were constructed separately for each of the three reproductive traits (marking rate, reproductive rate and lamb survival) for two maiden ewe age categories (maiden ewe lambs and two-tooth ewes) and two levels (pooled farm data and mob/paddock data), equating to 12 separate models. Ewe management during lambing (separate or combined management of maiden and multiparous ewes), ewe age, ewe breed, ram breed, month of mating, condition score at mating, feed-on-offer at mating, pasture type at mating, duration of supplementary feeding between mating and marking (at day 0–50, 50–100, 100–150 and lactation), supplementary feeding method (trail feeding or self-feeder), type of supplementary feed, condition score at lambing, average feed-on-offer and management category at lambing (mixed or separate management of single- and multiple-bearing ewes) were fitted as fixed effects. Mob pregnancy status (mob scanned as single, twin, triplet or mixed litter size) was fitted as a fixed effect at mob level. Year of lambing and farm were fitted as random effects. ABARE region was fitted as a random effect but was not significant and was subsequently removed from the final models. For all analyses, main effects and interactions were only included if they were statistically significant (*p* < 0.05).

## 3. Results

### 3.1. Characteristics of Survey Participants

A total of 79 respondents provided complete data for maiden and multiparous ewes and were eligible for inclusion in the study. Of these, 16 producers contributed data for two years, and three producers contributed data for three years to give a total of 103 survey responses that represented 111,117 maiden ewes managed in 307 mobs from lambing to marking (Table 1). A total of 302,585 multiparous ewes were included in eligible survey responses (Table 1).

Respondents were located across five Australian states (Table 1). The mean farm size was 3750 hectares (range: 230–115,000 hectares), and the mean number of breeding ewes per farm was 4762 (range: 477–25,000 ewes).

Characteristics for the management of sheep are shown in Table 2. Maiden Merino ewes were joined to Merino rams as two-tooth ewes to lamb at approximately two years-of-age with mean recorded age at mating of 18.5 months. The non-Merino ewe lambs included a range of different breeds, such as composite, Suffolk and Dorper. These were joined with maternal or terminal non-Merino rams to lamb at approximately one year of age with a mean age at mating of 8 months. Differential management during lambing based on ewe litter size was used by 47% of respondents (66% ewes) for maiden ewe lambs and 57% of respondents (61% ewes) for maiden Merino two-tooth ewes (Table 2).

The mean length of the mating period for maiden ewes was 39 days (Table 3). The condition score prior to lambing was reported by 75 respondents (Table 3), of which 31 (41%) were based on direct measurement, 35 (47%) were based on estimation and 9 (12%) did not specify the method used. The mean reported condition score prior to lambing for all maiden ewes was 3.1 (Table 3). Feed-on-offer during lambing was reported by only eight respondents, of which three were based on measurement, four were estimated and one did not specify the method used. Eighty-six per cent (*n* = 68) of respondents reported having been trained in the assessment of feed-on-offer and condition scoring.

### 3.2. Reproductive Performance in Maiden and Multiparous Ewes

**Figure 1:**
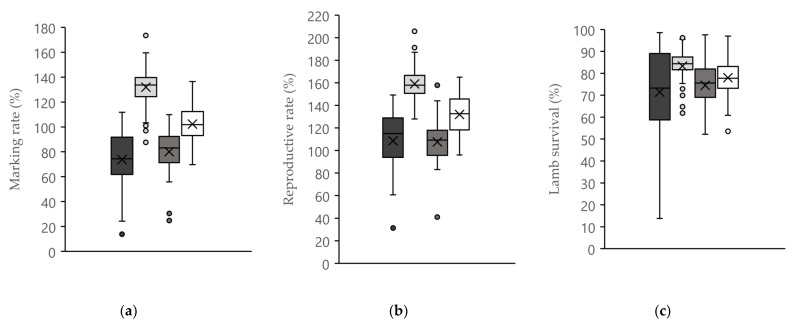
Box and whisker plot for (**a**) marking rate, (**b**) reproductive rate and (**c**) lamb survival in maiden ewe lambs, Merino two-tooth ewes and equivalent multiparous ewes. 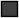 Maiden non-Merino ewe lambs. 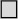 Multiparous non-Merino ewes. 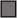 Maiden Merino two-tooth ewes. 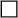 Multiparous Merino ewes.

Marking rate, reproductive rate and lamb survival for maiden and multiparous ewes are shown in Figure 1. A key difference between maiden ewe lambs and maiden Merino two-tooth ewes and both multiparous ewes was the wider variation in lamb survival and, to a lesser extent, reproductive rate and marking rate between flocks (Figure 1b).

Maiden ewes had a lower marking rate, reproductive rate and lamb survival compared to multiparous ewes on the same farm (Table 4). The average difference in marking rate between maiden and multiparous ewes was 58% for non-Merino ewe lambs and 22% for maiden Merino two-tooth ewes (Table 4). Lower marking rate in ewe lambs was attributable to differences of 51% for reproductive rate and 16% for lamb survival (Table 4). The poorer marking rates of maiden Merino two-tooth ewes compared with their multiparous counterparts was largely attributable to a 22% difference in reproductive rate, whilst the difference for lamb survival was only 3% (Table 3).

Ewe mortality of ewe lambs was not different to that of pregnant multiparous ewes (Table 4). However, the difference in mortality of Merino two-tooth ewes compared to their multiparous counterparts was 0.7% (Table 4).

There were moderate positive correlations between maiden Merino two-tooth ewes and their multiparous counterparts for marking rate, reproductive rate and lamb survival, marking rate and ewe survival (Table 5). In contrast, there was a very weak positive correlation between ewe lambs and their multiparous counterparts for lamb survival and no correlation for reproductive rate, marking rate or ewe survival (Table 5).

### 3.3. Factors Affecting Reproductive Performance of Maiden Ewes

Higher marking rates were observed for twin- and triplet-bearing ewes compared with single-bearing ewes (Table 6). This was due to the greater number of foetuses in these mobs and not due to a higher lamb survival with the difference in survival for twin lambs compared to single lambs of 11.4% for ewe lambs and 22.2% for Merino two-tooth ewes (Table 6 and Figure S1). Reproductive traits were independent of ABARE region, ewe age, ewe breed, ram breed, month of mating, condition score at mating, feed-on-offer at mating, pasture type at mating, condition score at lambing, duration of supplementary feeding, supplementary feeding method, type of supplementary feed or average feed-on-offer (*p* < 0.05).

## 4. Discussion

Maiden ewes mated as non-Merino ewe lambs or Merino two-tooth ewes produced 44 and 22 fewer lambs to marking per 100 ewes mated than their multiparous counterparts on the same farm. Lower marking rates for maiden compared to multiparous ewes was attributable, albeit to varying degrees, to lower reproductive rate and lower lamb survival. These results support our hypotheses. To our knowledge, this is the first study to compare the marking rate of maiden and multiparous ewes and their components on commercial farms across Australia. Development and adoption of management strategies to improve marking rate for non-Merino ewe lambs should focus on improving both reproductive rate and lamb survival as they contributed nearly equally to the differences in marking rate compared to multiparous ewes. By contrast, the poorer marking rate of Merino two-tooth ewes compared with their multiparous counterparts were largely due to differences in reproductive rate. Nevertheless, the development and adoption of strategies to improve marking rates for Merino two-tooth ewes should also focus on improving both reproductive rate and lamb survival. In this study, lamb survival was relatively low for both two-tooth and multiparous Merino ewes, suggesting that improved lamb survival for Merino ewes across all age groups remains an issue for the Australian sheep industry. Further, the economic value of improving reproductive rate is greater when lamb survival is higher [1], and improving lamb survival has implications for improving animal welfare [6].

The 58% difference in marking rate for non-Merino ewe lambs compared with multiparous ewes across 40 farms in our study was similar to the difference recently reported from nine commercial farms representing more than 300,000 records in New Zealand (72 vs. 142%) [14]. Likewise, marking rate was more variable between flocks for non-Merino ewe lambs compared with multiparous non-Merino ewes, with the overall average and variation in marking rate between flocks of non-Merino ewe lambs being similar to results reported by both Shorten et al. [14] and Clune et al. [9]. The 22% difference in marking rate for Merino two-tooth ewes compared to multiparous ewes across 39 farms was greater than the differences measured by Kleemann and Walker [10] on 14 farms in South Australia (70 vs. 86%) and a recent survey of 1200 Merino producers across Australia (79 vs. 93%) [15]. Over the last decade, more than 5000 sheep producers in Australia have participated in the extension and adoption programs to improve ewe management and reproductive performance, such as Lifetime Ewe Management [4,5] and Bred Well Fed Well [16], but these programs have only focused on the management of multiparous ewes. Similar extension and adoption programs to improve the reproductive performance of maiden ewes could have a significant impact on national marking rates and lamb supply as non-Merino ewe lambs and Merino two-tooth ewes represent approximately 10 million breeding ewes in Australia [3].

A lower marking rate for non-Merino ewe lambs compared to multiparous ewes on the same farm was attributed to a 51% difference in reproductive rate and a 16% difference in lamb survival. The difference in reproductive rate between these age groups was less than other studies, which have ranged from 75 to 124% [7,14], whereas the difference in lamb survival between these age groups tended to be greater than most other studies where the difference was less than 10% [14,17,18,19]. The wider variation in marking rate, reproductive rate and lamb survival between individual flocks of non-Merino ewe lambs was similar to these and other studies, including Clune et al. [9] and Thompson et al. (unpublished data). Improved understanding of the degree of variation and causes of poorer reproductive rate and lamb survival in maiden ewes will inform extension and adoption programs to improve maiden ewe reproductive performance.

The weak and generally non-significant correlation between the reproductive performance of ewe lambs and their multiparous counterparts in the current study hinged on the more variable performance of ewe lambs, whereas the performance of maiden two-tooth ewes was more consistent when compared with their multiparous counterparts on the same farm. Comparisons between studies suggest that the responses in reproductive rate to improved live weight at the start of the mating period and live weight gain during the mating period are much greater for ewe lambs than in multiparous ewes regardless of breed [8,9,20,21,22]. Recently Paganoni et al. [23] also reported that the effects of live weight and condition score at mating on reproductive rate were greater in ewe lambs compared to two-tooth non-Merino ewes within the same flocks, and the difference between these age groups was greater than the difference between two-tooth and multiparous Merino ewes. Age of mating also influences the reproductive rate of ewe lambs [8], even though this was not apparent in the current survey data where almost 90% of the ewe lambs were 7 or 8 months of age at mating. Collectively, this implies that the reproductive rate and potential marking rate of ewe lambs are more sensitive to management prior to and during mating than is the case for multiparous ewes. Bunter and Brown [24] reported that expression of reproduction at yearling and adult age is a genetically different trait in maternal (non-Merino) ewes. However, heritabilities for reproductive traits typically ranged 5–15%, which was consistent with low heritabilities for reproduction traits reported in other studies [25,26,27]. This indicates that whilst progress in reproductive traits can be made with selection, reproductive performance is driven mostly by management. Management strategies to improve the reproductive performance of ewe lambs are more complex and less adopted, which contributes to the poorer and more variable performance of maiden ewes compared to multiparous ewes on commercial farms.

The precise reasons for poorer and more variable survival of lambs born to maiden ewes, especially ewe lambs, compared with their multiparous counterparts were not able to be determined in this study. Maiden ewes were managed separately to multiparous ewes during pregnancy and lambing, and differences in the lambing environment, such as time of lambing, shelter and mob size, could have impacted lamb survival. Whilst the effects of the varying condition score at lambing on the survival of lambs born to two-tooth ewes is likely to be similar to that that observed in multiparous ewes [11], the slightly lower survival of lambs born to two-tooth ewes may reflect their average condition score at lambing, which was reported to be lower than recommended for mature ewes [28]. By contrast, whilst the level of nutrition and live weight change from pregnancy scanning to lambing is likely to influence the survival of lambs born to ewe lambs [29], the average condition score of ewe lambs in the current study was reported to be 3.2 at lambing, which is likely to be close to the optimum. It may, therefore, be that factors other than nutritional management during pregnancy and lambing contributed to the much greater difference in survival of lambs between ewe lambs and multiparous ewes compared to maiden two-tooth ewes and multiparous ewes.

A number of factors may contribute to the survival of lambs between scanning and marking. Important causes of perinatal lamb mortality include dystocia, stillbirths and starvation-mismothering, but these conditions are usually multifactorial and the role of dam parity are not fully understood [30,31,32,33]. Clune et al. [9] reported that lamb mortality between birth and marking was the major contributor to lamb loss between scanning and marking for Australian maiden ewe flocks, but that mid-pregnancy abortion caused significant in utero foetal loss in some flocks of ewe lambs. A review of veterinary investigations for abortions and stillbirths submitted to Australian veterinary diagnostic laboratories reported that 81% of investigations with a diagnosis involved infectious aetiology, with the most common infectious agents implicated being *Listeria* spp., *Campylobacter* spp. and *Toxoplasma gondii* [34]. More recently, *Chlamydia pecorum* has been associated with abortions and stillbirth for maiden ewe flocks in Australia [35,36]. A recent study reported *T. gondii, Neospora caninum* and *Coxiella burnetii* were not important contributors to foetal and lamb mortality maiden ewe flocks on farms in southern Australia [37,38,39]. Immunological naïvety is considered a risk factor for infectious reproductive diseases, and therefore maiden ewes have a higher risk for infectious causes of abortion because they have had less time to be exposed to infection and develop immunity before pregnancy [40,41,42,43]. Consequently, sporadic impacts of abortion and perinatal lamb mortality due to infectious disease could explain some of the variability in lamb survival reported in this study for maiden ewe flocks, and especially ewe lambs. Sporadic impacts of infectious disease on lamb survival could also explain the lack of correlation between lamb survival for ewe lambs and multiparous ewes on the same farm. The impacts of infectious disease on lamb survival for maiden ewes warrants further investigation.

We did not observe any effect of condition score or feed-on-offer on reproductive rate or lamb survival. However, there were very few responses that included data for condition score or feed-on-offer, and of these, a number were estimated rather than measured. Furthermore, condition score assessment is often subject to operator bias, which could result in between-operator variability in condition score assessment [44]. Other studies have reported associations between condition and reproductive rate and lamb survival, and between feed-on-offer at lambing and lamb survival. Therefore, the absence of effects of condition and feed-on-offer on reproductive traits in our study likely reflects the response numbers and reporting bias rather than the absence of a biological association. Further investigation to validate current recommendations for the condition at mating and during pregnancy for maiden ewes are warranted.

There were several limitations to this study. Surveys were distributed widely using electronic communications via a range of agricultural networks. As such, it was not possible to determine the survey response rate to provide an indication of non-responder bias. The most common differences between respondents and non-respondents cited in feedback were that non-respondents and responses not eligible for inclusion did not utilise pregnancy scanning to count foetuses, nor did they manage maiden and multiparous ewes separately at lambing to distinguish lamb survival and marking rates for both categories. The inclusion criteria generated bias in the sample population because only producers that utilised pregnancy scanning for litter size were eligible for inclusion. Subsequently, the sampled population likely included a higher proportion of ewes that were differentially managed according to litter size compared to the general population. Differential management of single- and multi-bearing ewes was used for 66% maiden non-Merino ewe lambs and 61% maiden Merino two-tooth ewes, which was consistent with the adoption rate for producers that had undertaken Lifetime Ewe Management training [5]. It is also possible that producers that have adopted pregnancy scanning were more likely to adopt other management strategies that could impact reproductive performance compared to the broader population. As such, the findings of this study should only be generalised to Australian sheep producers that have adopted pregnancy scanning and should not be extrapolated across the national sheep flock.

## 5. Conclusions

To our knowledge, this is the first study to compare the marking rate for maiden and multiparous ewes and their components on commercial farms across southern Australia. Marking rates for non-Merino ewe lambs and maiden Merino two-tooth ewes were lower than their multiparous counterparts, and this was attributable to a combination of lower reproductive rate and lower lamb survival. Wide variability in both reproductive rate and lamb survival indicates an opportunity for improvement in reproductive performance for maiden ewes through the adoption of strategies that increase the number of foetuses conceived and the number of lambs surviving between pregnancy scanning and lamb marking. Strategies specific to ewe lambs may be required because their reproductive performance was not correlated with multiparous ewes on the same farm.

## Figures and Tables

**Table 1:** Distribution of eligible survey responses by state based on number of respondents (farms) and total number of maiden and multiparous ewes.

			Ewes (*n*)				
	Respondents (*n*)		Maiden		Multiparous		
State	Non-Merino ewe lambs	Merino two-tooth ewes	Non-Merino ewe lambs	Merino two-tooth ewes	Non-Merino	Merino	**Total**
WA	3	11	2945	14,904	9580	37,305	**64,734**
SA	8	4	8974	4645	27,338	19,520	**60,477**
NSW	8	13	15,712	8818	38,216	29,226	**91,972**
VIC	19	11	34,387	13,728	80,448	43,931	**172,494**
TAS	2	0	7004	0	17,021	0	**24,025**
**Total**	**40**	**39**	**69,022**	**42,095**	**172,603**	**129,982**	**413,702**

**Table 2:** Maiden ewe management characteristics based on eligible survey responses showing number of responses and number of ewes managed by respondents.

	Ewe Lambs		Merino Two-Tooth Ewes	
	Responses (*n*)	Ewes (*n*)	Responses (*n*)	Ewes (*n*)
**Age at mating (months)**				
6	1	2545		
7	21	33,301		
8	25	28,379		
9	11	5003		
10	1	1361		
16			1	460
17			3	492
18			28	29,066
19			5	4898
20			3	2939
≥21			4	4213
**Month of mating**				
November			5	1770
December			7	4898
January	1	245	9	12,123
February	9	6188	11	8703
March	39	43,871	9	10,770
April	9	18,924	2	3614
May	1	1361	-	-
June			1	190
**Management during lambing**				
Mixed	30	23,574	19	16,575
Differential management ^A^	27	46,605	25	25,493
**Rams used for mating**				
Maternal	35	37,944		
Terminal	22	32,235		
Merino			44	42,068
**Supplementary feeding during lambing**				
Trail feeding	0	0	6	5656
Self-feeder	2	2753	3	1861
No supplementary feeding	30	50,027	17	16,576
Not indicated	25	17,399	17	17,289

^A^ Ewes scanned with single and multiple fetuses were managed separately during lambing.

**Table 3:** Characteristics for management of maiden ewes based on eligible survey responses.

	Ewe Lamb Flocks			Merino Two-Tooth Flocks		
	*n*	Mean ± s.e	Range	*n*	Mean ± s.e	Range
Mating period length (days)	43	40 ± 1	28–60	36	38 ± 1	28–56
Condition score						
Mating	47	3.15 ± 0.04	2.5–4.0	30	2.90 ± 0.06	2.0–3.7
Lambing	47	3.19 ± 0.05	2.7–4.0	28	3.02 ± 0.07	2.5–4.0
Feed-on-offer during lambing (kg DM/Ha)	6	1700 ± 93	1500–2000	2	750 ± 250	500–1000

s.e: standard error. DM/Ha: dry matter per hectare.

**Table 4:** Comparisons between maiden and mature multiparous ewes for reproductive rate, marking rate and lamb survival with mean ± standard error, 95% confidence interval (95% CI) for the difference and non-parametric related samples Wilcoxon signed-rank test.

	Ewe Age Group Mean ± Standard Error		Difference	
	Maidens	Multiparous	Mean (95% CI)	*p*-Value
**Non-Merino ewe lambs**				
Marking rate (%) ^A^	73.8 ± 2.8	131.9 ± 2.0	−58.1 (−64.3, −51.9)	< 0.001
Reproductive rate (%) ^B^	108.6 ± 3.7	159.1 ± 1.9	−50.5 (−59.0, −42.1)	< 0.001
Lamb survival (%) ^C^	67.3 ± 1.4	83.4 ± 0.9	−16.0 (−18.8. −13.2)	< 0.001
Ewe mortality (%) ^D^	2.6 ± 0.2	2.8 ± 0.2	−0.2 (−0.8, 0.5)	0.378
**Merino two-tooth ewes**				
Marking rate (%) ^A^	80.1 ± 2.6	102.3 ± 2.2	−22.3 (−26.9, −17.7)	< 0.001
Reproductive rate (%) ^B^	107.6 ± 3.3	131.9 ± 2.7	−24.4 (−29.5, −19.2)	< 0.001
Lamb survival (%) ^C^	74.5 ± 1.6	77.7 ± 1.3	−3.1 (−5.8, −0.5)	0.026
Ewe mortality (%) ^D^	1.7 ± 0.2	2.4 ± 0.3	−0.7 (−1.2, −0.2)	0.006

^A^ Marking rate = lambs marked/ewes mated × 100. ^B^ Reproductive rate = fetuses scanned/ewe joined × 100. ^C^ Lamb survival = lambs marked (live)/fetuses scanned × 100. ^D^ Ewe mortality = ewe deaths between scanning and lamb marking (scanned pregnant)/ewes pregnant × 100.

**Table 5:** Linear regression and bivariate Pearson correlation (two-tailed) between reproductive traits in maiden ewes and corresponding measure for multiparous counterparts.

	Regression			Pearson Correlation	
	Intercept	Slope	R^2^	Correlation Co-Efficient	*p*-Value
**Multiparous ewes vs. non-Merino ewe lambs**					
Marking rate (%) ^A^	32.40	0.314	0.050	0.223	0.096
Reproductive rate (%) ^B^	118.29	−0.061	0.001	−0.032	0.814
Lamb survival (%) ^C^	29.55	0.460	0.079	0.280	0.035
Pregnant ewe mortality (%) ^D^	2.107	0.180	0.032	0.180	0.222
**Multiparous ewes vs. Merino two-tooth ewes**					
Marking rate (%) ^A^	14.85	0.637	0.292	0.541	<0.001
Reproductive rate (%) ^B^	4.21	0.783	0.426	0.652	<0.001
Lamb survival (%) ^C^	11.02	0.823	0.389	0.635	<0.001
Pregnant ewe mortality (%) ^D^	0.822	0.368	0.434	0.659	<0.001

^A^ Marking rate = lambs marked/ewes mated × 100. ^B^ Reproductive rate = fetuses scanned/ewe joined × 100. ^C^ Lamb survival = lambs marked (live)/fetuses scanned × 100. ^D^ Pregnant ewe mortality = ewe deaths between scanning and lamb marking (scanned pregnant)/ewes pregnant × 100.

**Table 6:** Predicted means ± standard error for the marking (%) and lamb survival (%) according to ewe management group during lambing for non-Merino ewe lambs and maiden Merino two-tooth ewes.

Management Group during Lambing	Marking %		Lamb Survival %	
	Ewe Lambs	Two-Tooth Ewes	Ewe Lambs	Two-Tooth Ewes
Mixed	97.3 ± 2.9	91.4 ± 5.0	NA	NA
Single	75.2 ± 2.3	83.3 ± 3.3	76.0 ± 1.6	82.7 ± 1.8
Twin	129.8 ± 2.3	122.0 ± 3.6	64.6 ± 1.6	60.5 ± 2.0
Triplet	146.0 ± 7.9	NA	43.7 ± 3.9	NA

NA: not available (not measured or no eligible responses).

## Data Availability

The datasets generated and/or analysed during the current study are not publicly available but are available from the corresponding author on reasonable request pending permission from the funding body (Meat and Livestock Australia) and Murdoch University.

## Supplementary Materials

The following are available online at https://www.mdpi.com/article/10.3390/ani12040513/s1, Figure S1 Box and whisker plot of lamb survival in single- and multiple-born progeny of maiden ewe lambs and maiden Merino two-tooth ewes managed according to litter size.
