# Supplementary material for: Lower Reproductive Rate and Lamb Survival Contribute to Lower Lamb Marking Rate in Maiden Ewes Compared to Multiparous Ewes
Source: Animals (Basel). 2022 Feb 18;12(4):513. doi: 10.3390/ani12040513 (PMC8868299; doi:10.3390/ani12040513)
Supplement: Supplementary file 1 [file animals-12-00513-s001.zip › animals-1577870-supplementary.pdf]

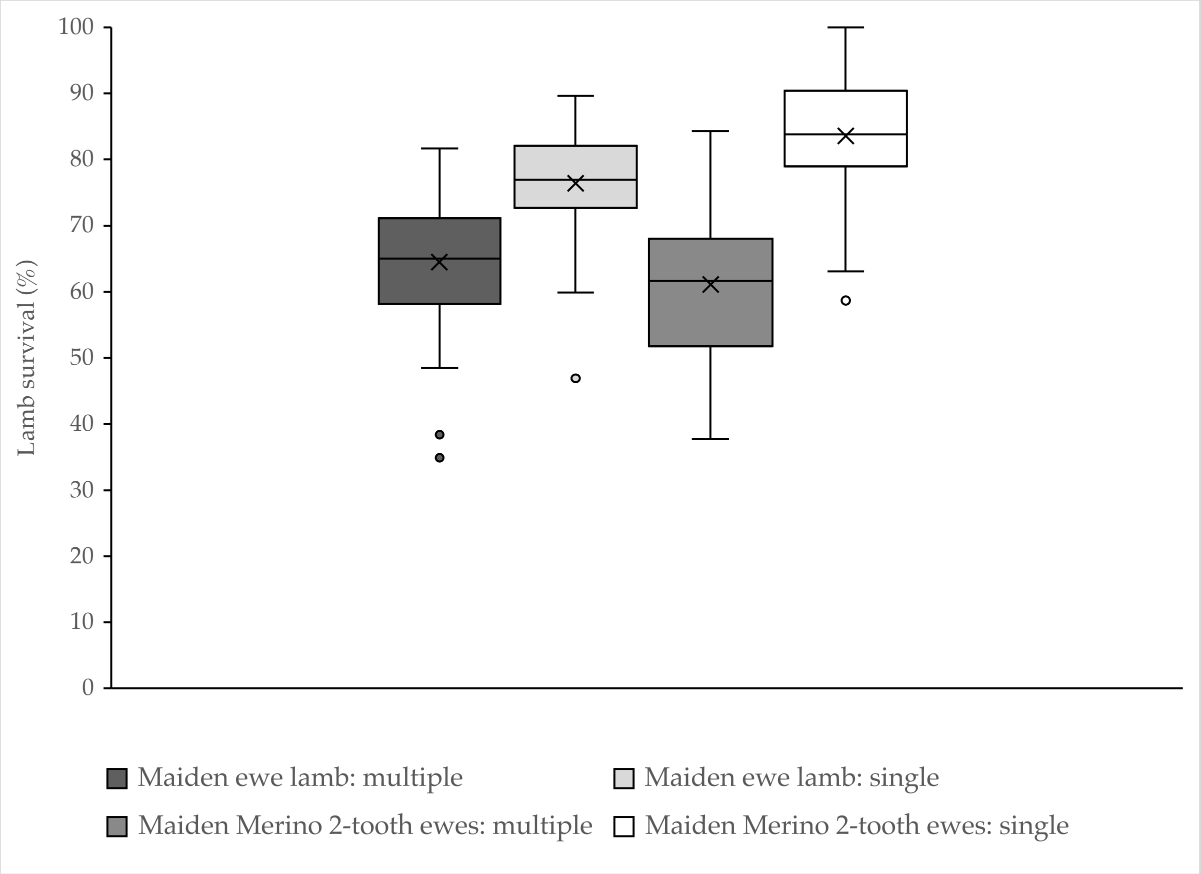

Figure S1. Box and whisker plot of for lamb survival in single- and multiple-born progeny of maiden ewe lambs and maiden Merino two-tooth ewes managed according to litter size
